# Antioxidant and Antimicrobial Activity of Rosemary (*Rosmarinus officinalis*) and Garlic (*Allium sativum*) Essential Oils and Chipotle Pepper Oleoresin (*Capsicum annum*) on Beef Hamburgers

**DOI:** 10.3390/foods11142018

**Published:** 2022-07-08

**Authors:** Paulina Olivas-Méndez, América Chávez-Martínez, Eduardo Santellano-Estrada, Luis Guerrero Asorey, Rogelio Sánchez-Vega, Ana Luisa Rentería-Monterrubio, David Chávez-Flores, Juan Manuel Tirado-Gallegos, Gerardo Méndez-Zamora

**Affiliations:** 1 UACH-CA03 Tecnología de Alimentos de Origen Animal, Facultad de Zootecnia y Ecología, Universidad Autónoma de Chihuahua, Periférico Fco. R. Almada, Chihuahua 33820, Mexico; zednem_pau@hotmail.com (P.O.-M.); amchavez@uach.mx (A.C.-M.); esantellano@uach.mx (E.S.-E.); rsanchezv@uach.mx (R.S.-V.); jtirado@uach.mx (J.M.T.-G.); 2 Food Technology Program, Institute of Agrifood Research and Technology (IRTA), Finca Camps i Armet, s/n, 17121 Monells, Spain; lluis.guerrero@irta.cat; 3 UACH-CA124 Química Aplicada y Educativa, Facultad de Ciencias Químicas, Universidad Autónoma de Chihuahua, Circuito Universitario s/n, Campus UACH II, Chihuahua 31125, Mexico; dchavezf@uach.mx; 4 Laboratorio de Ingeniería, Ingeniería en Industrias Alimentarias, Facultad de Agronomía, Universidad Autónoma de Nuevo León, Francisco Villa S/N, ExHacienda El Canadá, General Escobedo 66050, Mexico; mezage@hotmail.com

**Keywords:** lipid oxidation, antimicrobial activity, rosemary essential oil, garlic essential oil, chipotle pepper oleoresin, hamburgers

## Abstract

The inclusion of natural ingredients to preserve meat and meat products has increased in recent years. This study evaluated rosemary (REO) and garlic essential oils (GEO) as well as chipotle pepper oleoresin (CPO), alone or in combination, as preservatives on beef hamburgers (BH). Six treatments were evaluated: T1 (control, without additives), T2 (GEO 1%), T3 (REO 1%), T4 (CPO 0.5%), T5 (GEO 1% + CPO 0.5%) and T6 (REO 1% + CPO 0.5%). The microbiological quality, physicochemical characteristics, sensory evaluation, and lipid oxidation of hamburgers were evaluated. REO, GEO and CPO limited the growth of aerobic microorganisms, *S. aureus*, *Salmonella* spp., *B. thermosphacta,* moulds and yeasts, lactic acid bacteria and coliforms *(p <* 0.05); however, this effect depended on time. Furthermore, lipid oxidation decreased significantly (*p* < 0.5) in all treatments, except for T5 (GEO 1% + CPO 0.5%). Regarding sensory acceptance, consumers preferred BH with GEO in terms of colour, odour, flavour and overall appearance (*p* < 0.05). It is concluded that REO, GEO and CPO, alone or in combination, improve microbiological quality and inhibit the lipid oxidation of BH.

## 1. Introduction

BH and minced meat are among the most frequently consumed meat-based foods [1] although they are commonly associated with foodborne illnesses [2,3,4]. Spoilage of meat due to natural enzymatic processes and bacteria [5] is a significant problem within the food industry; however, the shelf life of meat products can be extended with natural or artificial preservatives [6]. The current trend tends towards natural biocides that are present in essential oils (EO) and chili oleoresins (CO) [7,8,9].

Essential oils are secondary products of plant and herb metabolisms, with antimicrobial and antioxidant properties, and are commonly used to enhance food’s flavour [10]. Approximately, from 3000 types of EO, 300 are used in the food industry [11,12].

The flavour, odour and colour of EO cause sensory changes in foods; hence, in meat and meat products the most used EO are those from oregano, rosemary, thyme, clove, garlic and basil [12,13].

Rosemary extracts and their essential oils have been used due to their antioxidant, antifungal and antimicrobial properties [14]. These properties are attributable to its chemical constituents such as rosmanol, carnosol, carnosic acid, ursolic acid, rosmariquinone, caffeic acid and rosmaridiphenol [15]. Furthermore, rosemary antioxidant properties are attributed to their high content of isoprenoid quinones, which act as chain terminators of free radicals and as chelators of reactive oxygen species [16]. These also inhibit the growth of *Escherichia coli*, *Bacillus cereus*, *Staphylococcus aureus* [17], *Clostridium perfringens*, *Salmonella choleraesuis, Brochothrix thermosphacta* and Enterobacteriaceae [18]. In addition, Bouloumpasi et al. [19] studied the antibacterial and antioxidant effects of rosemary by-products from the distillation of the essential oil on pathogenic and spoilage bacteria, concluding that they inhibit the growth of *Bacillus* (*B. subtilis*, *B. licheniformis*, *B. cereus*) strains and *Listeria monocytogenes*; they also found that the antioxidant activity of rosemary increased [19].

Regarding garlic essential oil (GEO), its antimicrobial activity is linked to its sulphur compounds [20]. Allicin has antimicrobial activity as it modifies lipid biosynthesis and profile as well as RNA synthesis of microorganisms [21]. It also inhibits more than 300 microorganisms, including Gram-positive and Gram-negative bacteria, acid-fast organisms and fungi. Michalczyk et al. [22] and Ibrahim-Hemmat et al. [23] found an inhibitory effect of GEO against *S. aureus*, *S. typhi*, *E. coli* and *L. monocytogenes* in meat products [22,23]. Furthermore, garlic contains sulphur compounds, selenium and free amino acids (especially cysteine, glutamine, isoleucine and methionine) to which it owes its antioxidant capacity [24]. Many of the active components found in garlic are effective in inhibiting the formation of free radicals and reinforcing the endogenous radical scavenging mechanism; they also increase cellular antioxidant enzymes, such as superoxide dismutase, catalase and glutathione peroxidase, and protect low-density lipoproteins from the oxidation caused by free radicals [8]. On the other hand, allicin acts as an antioxidant by reacting with enzymes that have free thiol groups, trapping free radicals, especially hydroxyl radicals. Amany et al. [25] found low values of malonaldehyde (MDA) in beef added to AEA, which indicated a high antioxidant capacity of the AEA components [25].

Furthermore, oleoresins are resin-like or viscous liquids extracted from plants with an organic solvent. These are used within the food and health industries because of their concentrated flavour and antioxidant properties. In the food sector, the most popular oleoresins are black pepper, garlic, oregano, rosemary, thyme and capsicum, among others [26,27,28]. Most oleoresins (solvent-free) are generally recognized as safe, as in the case of *Capsicum annuum* oleoresin [29].

*Capsicum* spp. is one of the most produced and consumed fruits (fresh, dry or oleoresin) wordwide due to its pungency, flavour and aroma [30,31]. It also has antimicrobial and antioxidant properties that can protect the food and the consumer from microbial and oxidative damage [32,33,34]. Mexico is considered the place of origin of *Capsicum annuum* (CA)*,* while other species are known to originate in South America [35]. These fruits contain capsaicinoids, which are responsible for its characteristic pungent taste. Capsaicinoids found in CA are 9–11 carbon chain branched fatty acid vanillylamides, and capsaicin and di-hydrocapsaicin are the most abundant [36], accounting for 90% of the total pungency of these fruits [37]. The most abundant capsaicinoids in CA oleoresins are capsaicin (48.6%), 6,7-dihydro capsaicin (36%), nordihydrocapsaicin (7.4%), homodihydrocapsaicin (2%), homocapsaicin (2%), capsanthin and capsorubicin. *Capsicum* oleoresins are recognized for their antimicrobial and antioxidant activities besides the colour and flavour they impart to foods [38].

Moreover, chipotle peppers are an important ingredient in Mexican cuisine and have become increasingly popular in Latin American gastronomy. The production of chipotle pepper is an artisanal process obtained by smoking matured jalapeño pepper (red jalapeño pepper) in open ovens, where firewood combustion gases are passed through for about 72 h [39]. Hence, their characteristic flavour and aroma come from the fruit and the smoking hardwood. Some of the chemical compounds found in them are guaiacol, formaldehyde, formic acid, acetone, short chain fatty acids, methanol, ethanol, furfural, acetaldehydes and volatile phenolic compounds [39]. In addition, it has been found that chipotle peppers have a higher antioxidant activity and content of bioactive compounds (phenols and carotenoids) than fresh peppers [40,41].

Several authors have reported the antimicrobial and antioxidant effects of EO and oleoresins on meat and meat products [42,43,44,45,46,47,48,49,50,51,52,53,54]. Al-Hijazen [54] showed that adding 150 ppm oregano EO + 350 ppm of rosemary extract to ground chicken had the highest antioxidant effect on lipids and proteins [54]. Furthermore, a study exploring the synergistic effect of EO, from herbs and spices, commonly used in meat products, reported that the EO from thyme, garlic, cumin and cinnamon have the lowest minimum inhibitory concentration (MIC); moreover, garlic essential oil inhibited the growth of *Salmonella* spp., *L. monocytogenes* and *S. aureus* [55]. In addition, rosemary extract power added to cured pork sausages reduced lipid oxidation [52]. Likewise, BH formulated with shirazi thyme, cinnamon and rosemary extracts had the lower degree of lipid and protein oxidation, as well as better scores on the sensory attributes of BH formulated with these extracts [46].

Although the antimicrobial and antioxidant properties of EO and oleoresins are well researched, few studies have addressed their combinations. To date, we have not found studies in BH evaluating the effect of adding CPO in combination with EO. Therefore, the aim of this study was to evaluate the effect of the addition of rosemary (REO, *Rosmarinus officinalis*) and garlic (GEO, *Allium sativum*) essential oils and chipotle pepper oleoresin (CPO, *Capsicum annum*) alone or in combination on the microbiological quality, lipid oxidation, physicochemical characteristics and sensory acceptance of BH.

## 2. Materials and Methods

### 2.1. Essential Oil and Oleoresin Extraction

The extracts were prepared from commercially available garlic, rosemary and chipotle pepper. The GEO, REO and CPO were extracted with organic solvents by stirring at a temperature of 150 °C (CPO—60 min; GEO—24 h; REO—3 h). For the extraction of GEO and CPO, ethanol was added at 70 (Duran et al., 2007) and 80%, respectively [56,57], and cyclohexane at 96% for the REO. Subsequently, the samples were filtered and solvents were removed with a rotary evaporator. For GEO and CPO, a temperature of 60 °C was used, and for REO it was 40 °C.

### 2.2. Treatment Description and Hamburger Preparation

Six treatments were prepared following a completely randomised design. Each treatment was performed in triplicate as follows: T1 (control, without additives), T2 (GEO 1% *w*/*w*), T3 (REO 1% *w*/*w*), T4 (CPO 0.5% *w*/*w*), T5 (GEO 1% + CPO 0.5% *w*/*w*) and T6 (REO 1% + CPO 0.5% *w*/*w*). The concentrations used were selected based on a previous sensory evaluation test (data not shown). Salt, water and fresh beef from *Semimembranosus* muscle (24 h *postmortem*) were used to prepare the base mixture as follows. First, the meat was frozen (−5 °C) and ground for 5 min (Torrey mill, M-12-FS, and 1/8” burger grind plate CI-12-18). Then, it was mixed with a salt/water solution (100 g of meat: 10 mL of water: 1 g of salt). Later, the mixture was divided equally into six parts, one per each treatment. After each treatment was prepared, hamburgers (150 g) were moulded and packed on polyethylene bags and stored aerobically at 4 °C. All treatments were analysed in triplicates at days 1, 8 and 15, except for the physicochemical and sensory evaluations that were assessed only at day 1.

### 2.3. Physicochemical Analyses

Physicochemical determinations were evaluated as follows: protein (AOAC 981.10), fat (AOAC 2007.04), ashes (AOAC 920.153) and moisture (AOAC 2007.04) [58]. The pH was determined with a potentiometer (Orion Versa Star, Thermo Scientific^®^, Singapore) [59]. Colour was evaluated in terms of CIELAB parameters, L* (whiteness or brightness), a* (redness or greenness) and b* (yellows or blueness), with a spectrophotometer (Chroma meter, Konica Minolta, CR-410, Japan), and the differences in colour (ΔΕ*) were calculated according to the following formula [60]:

$$∆E=\sqrt{{\left(L^{*}-L_{ref}^{*}\right)}^{2}+{\left(a^{*}-a_{ref}^{*}\right)}^{2}+{\left(b^{*}-b_{ref}^{*}\right)}^{2}}$$

where, L*_ref_, a*_ref_ and b*_ref_ = control parameters and L*, a* and b* = parameters for the different treatments.

### 2.4. Lipid Oxidation

Lipid oxidation was determined by the quantification of thiobarbituric acid reactive substances (TBARS) according to Pfalzgraf et al. [61]. Briefly, 10 g of meat was homogenized with 20 mL of trichloroacetic acid (10%), and then the homogenate was centrifuged and the supernatant decanted. Next, 2 mL of the filtrate was mixed with 2 mL of the TBA reagent (20 mM). The mixture was kept in a bain-marie for 20 min at 80 °C and after that, the absorbance was measured in a spectrophotometer at 531 nm. A standard calibration curve was developed with an increasing concentration of 1,1,3,3, tetraethoxypropane (4.73 mM, from 0 to 30 μL) (y = 144.2x + 0.0066, R^2^ = 0.9977). TBARS values were expressed as milligrams of malondialdehyde (MDA) per kilogram of muscle [61].

### 2.5. Microbiological Analyses

Ten grams from each treatment were aseptically homogenised in 90 mL of Maximum Recovery Diluent (MRD, CM0733, Oxoid©, Basingstoke, UK) and mixed in a Stomacher^®^ (Lab Blender, Seward, London, UK) at a maximum speed for 2 min. The homogenised sample was serially diluted (1:10) in MRD (CM0733, Oxoid©, Basingstoke, UK) according to the Official Mexican Standards. Each dilution (100 µL) was surface-plated onto specific media and incubated aerobically at 32 °C except for *B. thermosphacta* and moulds and yeasts, which were incubated at 25 °C as follows: total aerobic count (TAC, AOAC 990.12) on enriched Plate Count agar (PCA, CM0325, Oxoid©, Basingstoke, UK), *Staphylococcus aureus* (AOAC 2003.07) on Baird Parker agar (BP, 11723503, BD BIOXON^®^, Cuautitlan Izcalli, Mexico) with egg yolk and tellurite (S1058JAA, BD Difco^®^, Cuautitlán Izcalli, Mexico), total coliforms (AOAC 991.14) in Red Bile Violet agar (RBV, 70188, Fluka, Spruce, USA), moulds and yeasts (AOAC 997.02) in Potato Dextrose agar (PDA; 213300, BD BIOXON^®^, Heidelberg, Germany) acidified with 10% tartaric acid (T400 DL-tartaric, Merck, Saint Louis, MO, USA), *Salmonella* (NOM-114-SSA1-1994) [62] in Xylose, Lysine and Tergitol 4 agar (XLT4, R459802, Remel^®^, San Diego, USA), *Brochothrix thermopsphacta* (AOAC 303–306) in Streptomycin Thallium Acetate Actidione agar (STAA, CM0881, Oxoid©, Basingstoke, UK), lactic acid bacteria (LAB, NOM-243-SSA1-2010) [63] on de Man, Rogosa and Sharpe agar (MRS, 110660, MERCK^®^, Darmstadt, Germany) and *Pseudomonas* spp. (ISO 13720-2011) on Cetrimide, Fucidin, Cephalosporin agar (CFC, CM0559, Oxoid©, Basingstoke, UK) enriched with Supplement SR0103E (Oxoid©, Basingstoke, UK). The plates were incubated for the following times: RVB and BP 24 h, STAA and CFC 48 h, PCA 72 h and PDA and MRS 120 h [64]. Numbers of colony-forming units (CFU) were counted on plates with numbers ranging between 10 and 200 CFU and results were transformed from CFU/g to log_10_ CFU/g.

### 2.6. Sensory Evaluation

Sensory evaluation was conducted with an untrained panel of 50 consumers. According to Stone and Sidel [65], a panel of 25–50 subjects per product in laboratory testing is recommended [65]. Individuals between 18 and 50 years of age evaluated the BH sensorially through an acceptability test (colour, flavour, odour and general appearance) [66] using a 5-point hedonic scale (1: I dislike it a lot; 2: I dislike it little; 3: I don’t like it or dislike it; 4: I like it; 5: I like it a lot) according to Anzaldúa-Morales (1994) [67]. All samples were identified with a 3-digit code and were presented randomly to panellists. Panellists were indicated to rinse their palate with water between samples.

### 2.7. Statistical Analysis

A completely randomized one-way design was used to elaborate the treatments. Response variables were physicochemical composition (moisture, ashes, fat and protein), colour (L*, a*, b*, ΔE*), microbiological quality (*S. aureus*, *Salmonella* spp., *Pseudomonas* spp., *B. thermosphacta*, moulds and yeasts, lactic acid bacteria and total coliforms) and sensory evaluation (odour, colour, taste and general appearance). Three repetitions were performed for each treatment. Data were analysed using the ANOVA procedure, using the General Lineal Model (GLM) procedure in SAS, version 9.1.3 (SAS Institute Inc. E. U. A., 2006) [68]. Subsequently, a multiple comparison of means was carried out by the Tukey test, using a value of α = 0.05. Regarding the sensory evaluation, a correspondence analysis using the CORRESP procedure of the same statistical package (SAS version 9.1.3.) was performed to review which treatments corresponded more with the levels of sensory response on the hedonic scale.

The physicochemical composition (moisture, ashes, fat and protein) was analysed to compare means with a significance of *p* < 0.05 with the model:

y_ij_ = μ + τ_i_ + ε_ij_

where y_ij_ is the responding variable measured in the j-th repetition of the i-th treatment, τ_i_ is the effect of the i-th treatment and ε_ij_ is the random error corresponding to the j-th repetition of the i-th treatment.

Furthermore, colour, pH, lipid oxidation and antimicrobial activity were evaluated through time with the model:

y_ijk_ = μ + τ_i_ + P_j_ + τP_(ij)_ +ε_ijk_

where y_ij_ is the responding variable measured in the k-th repetition of the i-th treatment in the i-th period, τ_i_ is the effect of the i-th treatment, P_j_ is the effect of j-th period and τP_(ij)_ is the effect of the interaction of the i-th treatment with the j-th period and ε_ij_ is the random error corresponding to the k-th repetition of the i-th treatment in the i-th period.

## 3. Results and Discussion

### 3.1. Physicochemical Analyses

There were not statistically significant differences (*p* > 0.05) on the physicochemical composition among treatments (Table 1). The protein content on the BH ranged from 20.63 ± 0.87 to 21.74 ± 1.32; fat, from 10.63 ± 0.5 to 11.11 ± 1.07; moisture, from 70.62 ± 1.06 to 71.69 ± 1.23 and ashes from 2.22 ± 0.35 to 2.44 ± 0.14. Hence, the chemical composition of the hamburgers was within ranges reported previously [69,70]. It has been reported before that EO and CO do not affect the chemical composition of meat and meat products [22,71,72].

**Figure 1:**
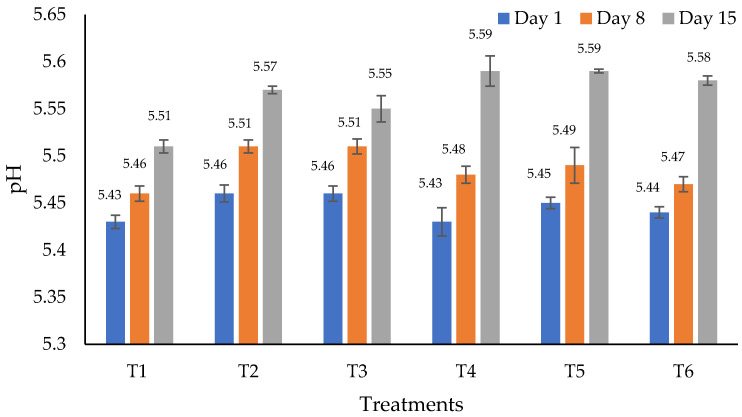
pH values (mean) of beef hamburgers added to essential oils of rosemary (*Rosmarinus officinalis*) and garlic (*Allium sativum*) and oleoresins of chipotle pepper (*Capsicum annum*). T1 = control, T2 = 1% garlic essential oil (*w*/*w*), T3 = 1% rosemary essential oil (*w*/*w*), T4 = 0.5% chipotle pepper oleoresin (*w*/*w*), T5 = 1% garlic essential oil + 0.5% chipotle pepper oleoresin (*w*/*w*) and T6 = 1% rosemary essential oil + 0.5% chipotle pepper oleoresin (*w*/*w*). There was no difference between treatments (*p* > 0.05).

Figure 1 shows the pH values among treatments through time. There were not significant (*p* > 0.05) differences in pH among treatments either through time (*p* > 0.05); values ranged from 5.43 ± 0.007 to 5.59 ± 0.086. These values were within a range of 5.3 to 5.6 which has been described as normal for meat hamburgers [73]. Likewise, similar results were reported in chicken when using EO as preservatives [74].

### 3.2. Colour

Table 2 shows colour parameters: L*, a*, b* and colour difference (ΔE*). Luminosity (L*) showed significant changes through time (*p* < 0.05) but no differences among treatments (*p* > 0.05). All treatments had greater values on days 8 and 15 compared to day 1 (*p* < 0.05). L* indicates the degree of brightness of a colour, ranging from 0 (black) to 100 (white), and it is related to the content of pigments in a food [75]. A food with greater content of pigments has a stronger light absorption, resulting in a lower reflectance; hence, the food can be darker or opaquer. In meat, colour is related to levels of myoglobin and the relative proportions of each redox form [76]. As mentioned before, all treatments showed a lower L* at day 1 (*p* < 0.05), meaning these treatments had greater concentrations of pigments at this day. Then, L* increased at days 8 and 15 without statistically significant differences among them (*p* > 0.05). Hernández et al. [76] measured the colour of meat samples at 1, 4 and 7 days of exposure to air and found significant differences in the L* coordinate between days 1 and 4, but not between days 4 and 7 [76]. They stated that there is a reasonably direct relationship between total pigment content and sample lightness.

If the value of a* is positive, it indicates a tendency to red, and if it is negative, to green. In terms of a*, significant differences were found among treatments and overtime (*p* < 0.05). At day 1, T4 presented the greatest value (13.29 ± 1.15, *p* < 0.05) and no significant differences were found among the rest of the treatments (*p* > 0.05). At day 8, treatments 4 and 6 had the greatest values compared with the other treatments (*p* < 0.05, 10.89 ± 1.32 and 10.28 ± 1.02, respectively), without significant differences among them (*p* > 0.05). Finally, on day 15, T4 and T6 presented the greatest values 12.30 ± 0.73 and 11.80 ± 1.16, respectively, without statistically significant differences among them (*p* > 0.05). The latter could be associated with the carotenoids and capsaicinoids of the CPO [77]; carotenoids are tetraterpenoid pigments that present chromophore properties. CPO also has capsaicinoids such as capsaicin, which is a red natural pigment and is the predominant capsaicinoid presented in chili plants [56]. The lowest values of a* through time were found on day 8 in all treatments (*p* < 0.05).

Myoglobin is a water-soluble protein that contains iron in its structure. In fresh meat, it is mainly found in three basic states: deoxymyoglobin (DMb), oxymyoglobin (OMb) and metmyoglobin (MMb) [76]. Oxymyoglobin gives the bright red colour to meat which is visually and colourimetrically redder than DMb and MMb; therefore, the presence of OMb influences the value of a* [76]. In the presence of oxygen, myoglobin oxidizes to OMb; however, this oxidized form can undergo a deoxygenation process under an atmosphere with low oxygen tensions, which can be promoted by the presence of salt [78]. The above could explain the non-linear relationship of a* with respect to time. This non-linear behaviour was also observed in frozen BH added to butilhidroxitolueno (100 ppm) during storage at 30 days [46].

In terms of b* (positive values indicate a tendency to yellow and negative values a tendency to blue), no significant differences were observed among treatments (*p* > 0.05). The lowest values of b* through time were found in day 1 in all the treatments (*p* < 0.05).

Finally, colour differences (∆E*) were not significantly different (*p* < 0.05) through time. However, significant differences were found among treatments (*p* < 0.05). Compared to T4, T5 and T6, ∆E* in T2 and T3 were lower (*p* < 0.05). Treatments 2 and 3 do not contain CPO in their formulations. According to Ramírez-Navas and Stouvenel, ΔE* greater than 2.7 makes the colour difference noticeable to the human eye [60]. Therefore, ΔE* presented in T4, T5 and T6 would be appreciated. These colour changes could be associated with the content of the carotenoids and capsaicinoids of the CPO. As said before, carotenoids are terpenoid pigments with various terminal groups which cause varied chromophore properties, such as the red colour captured by the human eye [77]. CPO also contains capsaicinoids in its structure, the most important being hydrocapsaicin and capsaicin, the latter being the most predominant capsaicinoid as well as the natural red pigment found in chili plants [56].

### 3.3. Lipid Oxidation

**Figure 2:**
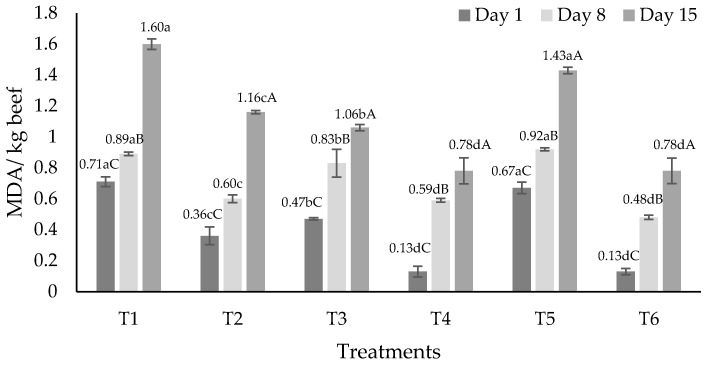
Lipid oxidation (MDA/kg beef, mean) over time in beef hamburgers added to rosemary (*Rosmarinus officinalis*) and garlic (*Allium sativum*) essential oils and chipotle pepper (*Capsicum annum*). T1 = control, T2 = 1% garlic essential oil (*w*/*w*), T3 = 1% rosemary essential oil (*w*/*w*), T4 = 0.5% chipotle pepper oleoresin (*w*/*w*), T5 = 1% garlic essential oil + 0.5% chipotle pepper oleoresin (*w*/*w*) and T6 = 1% rosemary essential oil + 0.5% chipotle pepper oleoresin (*w*/*w*). ^a,b,c^ = different lowercase literals indicate significant difference (*p* < 0.05) between treatments. ^A,B,C^ = different capitalized literals indicate significant difference (*p* < 0.05) through time.

In all treatments, lipid oxidation varied (*p* < 0.05) throughout time (Figure 2). Being observed, the lowest values were on day 1 compared to days 8 and 15. Among treatments, on days 1, 8 and 15, T4 and T6 presented the lowest values (*p* < 0.05) and T1 and T5 had the greatest values (*p* < 0.05). On day 1, values for T4 and T6 were 0.13 ± 0.035 and 0.13 ± 0.02, respectively, and values for T1 and T5 were 0.71 ± 0.032 and 0.67 ± 0.037, respectively. Lipid oxidation was less in treatments that had CPO or CPO in combination with REO (T4 and T6). This stability may be associated with the antioxidant capacity of the chemical components present in REO and CPO. *Capsicum* fruits, and consequently CPO, have an antioxidant capacity due to their capsaicinoids, mainly capsaicin and dihydrocapsaicin [79]. Capsaicinoids react with the free radicals of meat [80] and reduce lipid oxidation in aerobic conditions [81]. Furthermore, *Capsicum* peppers have vitamin E [82,83] that acts as an electron-donor antioxidant [84], and rosemary is among the plants with higher antioxidant concentration [85]. The antioxidant effect in rosemary is associated with the content of carnosol, carnosic acid, rosmanol, rosmariquinone and rosmaridiphenol which acts as hydrogen atom donors [84]. Carnosic acid and carnosol, combined with compounds that share the same chemical structure, may have a synergetic effect that chelate oxygen reactive species [86]. Hence, the combination of REO and CPO in T6 may cause a synergistic effect; such effects have been reported before in meat [84,87]. Carotenoids may have an antagonistic effect in the presence of other antioxidants or in aerobic conditions [88], and this could have occurred with the combination of GEO and CPO in T5.

### 3.4. Antimicrobial Analyses

Total aerobic bacteria increased over time in all the treatments (Table 3). Days 1 and 8, in all treatments, presented lower counts compared with day 15 (*p* < 0.05). Among treatments, in all days, T1 presented higher counts (*p* < 0.05).

Counts of *S. aureus* (Table 3) at day 1 and 8 were not different among treatments (*p* > 0.05). In all treatments, the numbers of *S. aureus* increased from day 1 to 8 (*p* < 0.05), and from day 8 to 15, in T1 and T4 counts decreased (*p* < 0.05) and in T2, T3, T5 *S. aureus* was not detected. *Salmonella* spp. was not detected on any day and in any treatment. In terms of *Pseudomonas* spp., days 1 and 8, in all treatments, presented lower counts compared to day 15 (*p* < 0.05). Among treatments, in all days, T1 presented higher counts (*p* < 0.05) compared to the other treatments. With respect to *B. thermosphacta*, in day 1, this was not detected in any treatments. Moreover, on day 7 it was only counted in T1 and T3, being greater for T1 (1.99 ± 0.11 and 1.54 ± 0.82, respectively) (*p* < 0.05). Finally, on day 15, only T1, T2, T4 and T5 presented counts, being also greater for T1 (*p* < 0.05). With respect to moulds and yeast, in all treatments, they were not detected on days 1 and 8. Moreover, on day 15, T1 had higher counts (*p* < 0.05) compared to T2, T3 and T4. In addition, no significant differences were found among T1, T5 and T6 (*p* > 0.05). Regarding LAB, they were not detected on day 1 on T2, T3, T5 and T6, and T1 and T4 had similar counts (*p* > 0.05). On day 8, only T1, T2 and T5 presented counts without differences among them (*p* > 0.05). Finally, on day 15, all treatments presented counts, T3 with the lowest counts (*p* < 0.05). Coliforms were not detected on days 1 and 8 in all treatments, and on day 15 they only were detected on T1 and T3, 0.77 ± 0.70 and 0.43 ± 0.73, respectively, without differences among them (*p* > 0.05).

The role of GEO, REO and CPO as food preservatives, due to their antimicrobial effect against pathogens and spoilage microorganisms, such as *S. aureus*, P*seudomonas*, *B. thermosphacta* and *Salmonella*, is well documented [18,23,89]. GEO has a great variety of compounds, such as enzymes, amino acids and minerals, that contribute to its antimicrobial activity [90]. However, the most biological active ones are the sulphur compounds and their precursors such as allicin, ajoene and diallyl disulphide, among others [91]. Sulphur compounds react with the cysteine and inhibit thiol-containing enzymes (proteases and alcohol dehydrogenase) or the metabolism of lipids [92]. These compounds also inhibit microbial respiration and modify the synthesis of RNA [21], as well as increase the permeability of the plasmatic membrane, which results in cellular death after a massive leak of ions [17,93,94].

The main components of REO are terpenes and terpenoids [95]. The lipophilic nature of terpenes and terpenoids allows them to disrupt the lipopolysaccharide chain in the membrane, and if the damage is large enough it can reach the cytoplasm and interrupt vital cell functions [96]. Moreover, the OH groups in their structure might be toxic for some bacteria.

*Capsicum* fruits and their extracts, such as CPO, have capsaicin and dihydrocapsaicin. These compounds distort and disrupt the structure and functionality of the cytoplasmatic membrane [97], mainly in Gram-positive, as the effect is more peptidoglycan-specific [98]. The latter might explain why treatments 4, 5 and 6 inhibited *S. aureus, B. thermosphacta* and LAB. Finally, there is evidence of a synergetic effect between some essential oils and chili oleoresins [9,99], hence showing a greater antimicrobial activity, as was observed in T5 and T6.

However, in this study, GEO, REO and CPO used alone or in combination had no effect in reducing *S. aureus* at days 1 and 8 (*p* > 0.05), and for *Pseudomonas* spp, only T3 at day 1 and T2 at day 8 had significant lower counts (*p* < 0.05).

This could be since there are several factors that affect the antimicrobial properties of essential oils. In a food, EO interact with the constituents of the food (fat, proteins, starch, etc.) [93]. Moreover, the antimicrobial effect of the EO constituents also depends on pH, temperature and the level of microbial contamination [93]. Then the effectiveness of EO is greater in a culture media than in foods. In meat products, the content of fat has a great effect on the antimicrobial effectiveness of EO against most microorganisms. Because of this, the concentration and/or quantity of EO used as a preservative in foods should be higher than the one used in in vitro conditions. However, the sensory acceptance of food products may limit the quantity of the EO that can be added. For this, different strategies should be explored to circumvent this problem.

### 3.5. Sensory Evaluation

**Figure 3:**
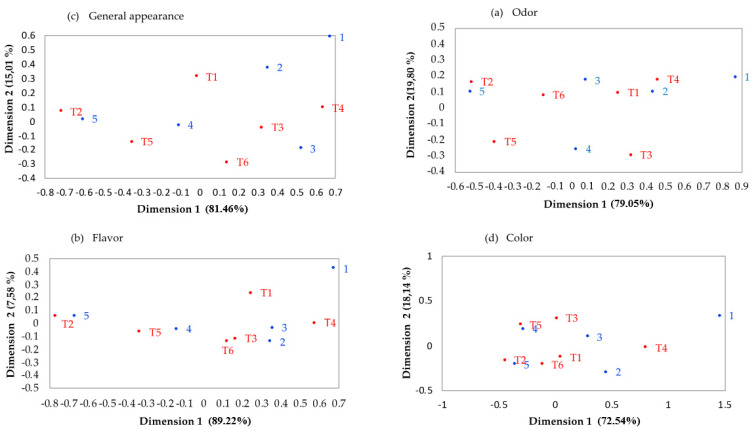
Correspondence analysis of general appearance (**c**), odour (**a**), flavour (**b**) and colour (**d**) of beef hamburgers added to rosemary (*Rosmarinus officinalis*) and garlic (*Allium sativum*) essential oils and chipotle pepper (*Capsicum annum*). T1 = control, T2 = 1% garlic essential oil (*w*/*w*), T3 = 1% rosemary essential oil (*w*/*w*), T4 = 0.5% chipotle pepper oleoresin (*w*/*w*), T5 = 1% garlic essential oil + 0.5% chipotle pepper oleoresin (*w*/*w*) and T6 = 1% rosemary essential oil + 0.5% chipotle pepper oleoresin (*w*/*w*); 1: I dislike it a lot; 2: I dislike it little; 3: I don’t like it or dislike it; 4: I like it; 5: I like it a lot.

In general appearance (Figure 3c) (Figure 3a), it was observed that T2 was preferred by panellists because it had the highest correspondence with level 5 (I really like it), T1, T5 and T6 had higher correspondence with level 4 (I like it) and T3 and T4 had correspondence with level 3 (neither I like nor dislike). A correspondence analysis of odour is shown in Figure 3a, T2 and T5 correspond to level 5 (I like it a lot), T4 and T1 correspond to level 2 (I dislike it little), T6 corresponds to level 3 (neither I like nor dislike) and T3 correspond to level 4 (I like it). A flavour correspondence analysis is presented in Figure 3b, where it is observed that T2 corresponds to level 5 (I really like it), T5 corresponds to level 4 (I like it), T4 as well as T1 correspond to level 3 (I don’t like or dislike it) and T6 and T3 correspond to level 2 (I dislike it a little). Finally, regarding the correspondence of colour (Figure 3d), T2 and T6 correspond to level 5 (I like it a lot), T5 corresponds to level 4 (I like it), T4 corresponds to level 2 (I dislike it a little) and T1 and T3 correspond to level 3 (neither I like nor dislike). Then, it can be inferred that treatments 2 and 5 were the best evaluated. These treatments are the ones with GEO and GEO/CPO. It has been reported that the addition of GEO modifies positively the acceptability and the sensory attributes of meat and meat products [100,101], while delaying off-flavours and off-odours associated with oxidation [102]. Our results showed that the addition of GEO, REO and CPO delayed lipid oxidation. It is worth mention that as many Mexican dishes are spiced with garlic and chipotle pepper, and these are among the most used spices in Mexican cuisine [103], these were chosen as preservatives for the BH.

Furthermore, the oleoresins of some chili plants rich in carotenoids and phenols have been described as shelf-life lengtheners in foods. At the same time, as these compounds join some meat components, they favour mechanisms that may improve sensory characteristics [104].

Low concentrations of essential oils, alone or in combination with other additives such as chili oleoresins, do not negatively alter the sensory attributes of meat [105] but improve the flavour, odour and colour of meat products [106,107].

## 4. Conclusions

The addition of GEO, REO and CPO to BH did not affect its chemical composition, pH or colour, but it reduced lipid oxidation. Moreover, regarding lipid oxidation, an antagonic effect was observed in the treatment containing GEO and CPO. Regarding microbial quality, the addition of GEO and CPO, alone or in combination, reduced aerobic bacteria and mould and yeast; however, the effect that GEO and CPO had against the rest of the microorganisms depended on the day and the treatments. Furthermore, consumers had greater acceptance for hamburgers formulated with GEO, REO and CPO. Because of these findings, it is concluded that GEO, REO and CPO can be used in BH formulations to improve microbial quality and avoid lipid oxidation.

## Figures and Tables

**Table 1:** Chemical composition (mean ± standard deviation) of beef hamburgers added to essential oils of rosemary (*Rosmarinus officinalis*) and garlic (*Allium sativum*) and oleoresins of chipotle pepper (*Capsicum annum*).

Treatments ^1^	Fat	Protein	Moisture	Ashes
T1	11.60 ± 0.21	21.74 ± 1.32	70.69 ± 1.52	2.44 ± 0.14
T2	11.06 ± 0.92	21.80 ± 0.89	70.62 ± 1.77	2.30 ± 0.34
T3	10.70 ± 1.19	21.29 ± 1.00	71.26 ± 1.00	2.36 ± 0.29
T4	11.11 ± 0.65	21.55 ± 0.96	71.62 ± 1.06	2.30 ± 0.17
T5	10.63 ± 0.50	20.63 ± 0.87	70.70 ± 1.35	2.29 ± 0.29
T6	11.11 ± 1.07	20.80 ± 0.99	71.69 ± 1.23	2.22 ± 0.35

^1^ T1 = control, T2 = 1% garlic essential oil (*w*/*w*), T3 = 1% rosemary essential oil (*w*/*w*), T4 = 0.5% chipotle pepper oleoresin (*w*/*w*), T5 = 1% garlic essential oil + 0.5% chipotle pepper oleoresin (*w*/*w*) and T6 = 1% rosemary essential oil + 0.5% chipotle pepper oleoresin (*w*/*w*). There was no difference between treatments (*p* > 0.05).

**Table 2:** Colour parameters (mean ± standard deviation) L*, a* and b* over time in beef hamburgers added to essential oils of rosemary (*Rosmarinus officinalis*), garlic (*Allium sativum*) and chipotle pepper (*Capsicum annum*) oleoresin.

Parameter	Time (d)	Treatments ^1^					
		T1	T2	T3	T4	T5	T6
L*	1	31.73 ± 1.19 ^a,B^	30.52 ± 1.43 ^a,B^	31.27 ± 1.43 ^a,B^	31.55 ± 1.75 ^a,B^	30.27 ± 1.36 ^a,B^	30.70 ± 1.14 ^a,B^
	8	32.34 ± 1.40 ^a,A^	32.57 ± 0.78 ^a,A^	32.00 ± 1.32 ^a,A^	31.68 ± 1.32 ^a,A^	31.98 ± 2.75 ^a,A^	32.13 ± 1.87 ^a,A^
	15	31.47 ± 1.04 ^a,A^	31.39 ± 1.14 ^a,A^	32.88 ± 0.78 ^a,A^	31.56 ± 0.96 ^a,A^	31.42 ± 0.86 ^a,A^	33.50 ± 0.77 ^a,A^
a*	1	12.17 ± 2.90 ^b,A^	11.62 ± 2.37 ^b,A^	10.55 ± 1.25 ^b,A^	13.29 ± 1.15 ^a,A^	11.37 ± 1.34 ^b,A^	11.53 ± 3.38 ^b,A^
	8	8.33 ± 1.34 ^b,B^	8.76 ± 1.08 ^b,B^	8.05 ± 1.59 ^b,B^	10.89 ± 1.32 ^a,B^	9.10 ± 1.32 ^b,B^	10.28 ± 1.02 ^ab,B^
	15	10.60 ± 1.02 ^b,A^	11.42 ± 0.81 ^b,A^	11.06 ± 0.80 ^b,A^	12.30 ± 0.73 ^a,A^	10.73 ± 0.52 ^b,A^	11.80 ± 1.16 ^ab,A^
b*	1	6.95 ± 0.56 ^a,B^	6.64 ± 0.59 ^a,B^	6.72 ± 0.56 ^a,B^	8.35 ± 1.04 ^a,B^	7.40 ± 0.60 ^a,B^	7.59 ± 0.74 ^a,B^
	8	10.66 ± 1.19 ^a,A^	10.82 ± 0.84 ^a,A^	10.52 ± 2.56 ^a,A^	10.60 ± 2.56 ^a,A^	11.11 ± 0.98 ^a,A^	11.60 ± 0.99 ^a,A^
	15	12.13 ± 0.95 ^a,A^	11.88 ± 1.00 ^a,A^	11.28 ± 1.32 ^a,A^	8.96 ± 1.66 ^a,A^	9.26 ± 0.41 ^a,A^	11.40 ± 0.76 ^a,A^
ΔE*	1	-	1.74 ± 0.57 ^b,A^	2.06 ± 0.87 ^b,A^	3.65 ± 1.16 ^a,A^	3.22 ± 1.16 ^a,A^	3.66 ± 1.55 ^a,A^
	8	-	1.80 ± 0.76 ^b,A^	2.28 ± 0.98 ^b,A^	3.72 ± 1.06 ^a,A^	3.06 ± 1.45 ^a,A^	3.42 ± 2.04 ^a,A^
	15	-	2.49 ± 1.14 ^b,A^	2.23 ± 0.91 ^b,A^	3.65 ± 1.45 ^a,A^	3.67 ± 0.48 ^a,A^	3.30 ± 1.47 ^a,A^

L* = luminosity, a* = green (−) and red (+), b* = blue (−) and yellow (+). ΔE* = colour difference calculated according to ΔE^*^ = √(L* − L*_ref_)^2^ + (a* − a*_ref_)^2^ + (b* − b*_ref_)^2^. ^1^ T1 = control, T2 = 1% garlic essential oil (*w*/*w*), T3 = 1% rosemary essential oil (*w*/*w*), T4 = 0.5% chipotle pepper oleoresin (*w*/*w*), T5 = 1% garlic essential oil + 0.5% chipotle pepper oleoresin (*w*/*w*) and T6 = 1% rosemary essential oil + 0.5% chipotle pepper oleoresin (*w*/*w*). ^a,b^ = different literals in the same row indicate significant difference (*p* < 0.05) between treatments. ^A,B^ = different literals in the same column indicate significant difference (*p* < 0.05) through time.

**Table 3:** Microbial quality (log_10_ CFU/g, mean ± standard deviation) of beef hamburgers added to rosemary (*Rosmarinus officinalis*) and garlic (*Allium sativum*) essential oils and chipotle pepper (*Capsicum annum*) over time.

Microorg	Time (d)	Treatment ^1^					
		T1	T2	T3	T4	T5	T6
TAC	1	2.59 ± 0.90 ^a,B^	1.82 ± 0.17 ^b,B^	1.60 ± 0.14 ^b,B^	1.82 ± 0.02 ^b,B^	1.75 ± 0.24 ^b,B^	1.58 ± 0.22 ^b,B^
	8	3.14 ± 0.02 ^a,B^	2.37 ± 0.28 ^b,B^	2.77 ± 0.11 ^b,B^	3.30 ± 0.47 ^b,B^	2.43 ± 0.12 ^b,B^	2.60 ± 0.17 ^b,B^
	15	5.25 ± 0.14 ^a,A^	4.48 ± 0.00 ^b,A^	4.7 ± 0.06 ^b,A^	4.8 ± 0.10 ^b,A^	4.52 ± 0.39 ^b,A^	4.35 ± 0.26 ^b,A^
*S. aureus*	1	1.26 ± 0.21 ^a,A^	1.31 ± 0.19 ^a,A^	1.08 ± 0.09 ^a,A^	0.85 ± 0.58 ^a,A^	1.01 ± 0.68 ^a,A^	0.60 ± 0.58 ^a,A^
	8	1.37 ± 0.27 ^a,B^	1.50 ± 0.01 ^a,B^	1.38 ± 0.09 ^a,B^	1.35 ± 0.11 ^a,B^	1.32 ± 0.09 ^a,B^	1.37 ± 0.25 ^a,B^
	15	1.02 ± 0.35 ^a,C^	ND	ND	0.79 ± 0.56 ^a,C^	ND	ND
*Salmonella* spp.	1	ND	ND	ND	ND	ND	ND
	8	ND	ND	ND	ND	ND	ND
	15	ND	ND	ND	ND	ND	ND
*Pseudomonas* spp.	1	1.16 ± 0.13 ^b,B^	1.06 ± 0.61 ^b,B^	0.37 ± 0.40 ^a,B^	1.10 ± 0.63 ^b,B^	1.00 ± 0.58 ^b,B^	1.00 ± 0.58 ^b,B^
	8	2.51 ± 0.08 ^a,B^	1.56 ± 0.03 ^b,B^	2.05 ± 0.23 ^b,A^	2.44 ± 0.33 ^b,B^	2.25 ± 0.49 ^b,B^	2.43 ± 0.36 ^b,B^
	15	3.25 ± 0.02 ^a,A^	2.17 ± 0.06 ^b,A^	2.68 ± 0.14 ^b,A^	2.51 ± 0.01 ^b,A^	2.28 ± 0.08 ^b,A^	2.58 ± 0.09 ^b,A^
B. therm	1	ND	ND	ND	ND	ND	ND
	8	1.99 ± 0.11 ^a,B^	ND	1.54 ± 0.82 ^b,B^	ND	ND	ND
	15	3.24 ± 0.13 ^a,A^	2.5 ± 0.08 ^b,A^	ND	0.27 ± 0.13 ^c,A^	0.50 ± 0.08 ^c,A^	ND
M&Y	1	ND	ND	ND	ND	ND	ND
	8	ND	ND	ND	ND	ND	ND
	15	1.24 ± 0.03 ^a,A^	0.52 ± 0.20 ^b,A^	0.30 ± 0.24 ^b,A^	0.22 ± 0.28 ^b,A^	1.07 ± 0.18 ^a,b,A^	0.82 ± 0.11 ^a,b,A^
LAB	1	0.6 ± 0.58 ^a,B^	ND	ND	0.60 ± 0.58 ^a,B^	ND	ND
	8	0.92 ± 0.78 ^a,B^	0.60 ± 0.58 ^a,B^	ND	ND	0.6 ± 0.58 ^a,B^	ND
	15	1.6 ± 1.02 ^a,A^	0.99 ± 0.67 ^a,b,A^	0.87 ± 0.30 ^b,A^	1.15 ± 0.35 ^a,b,A^	0.85 ± 0.58 ^b,A^	1.22 ± 0.20 ^a,b,A^
Total coliforms	1	ND	ND	ND	ND	ND	ND
	8	ND	ND	ND	ND	ND	ND
	15	0.77 ± 0.70 ^a,A^	ND	0.43 ± 0.73 ^a,A^	ND	ND	ND

CFU = colony-forming unit; Microorg = microorganism; d = day; TAC = total aerobic count; *S. aureus = Staphylococcus aureus*; B. therm = *Brochothrix thermosphacta*; M&Y = moulds and yeasts; LAB = lactic acid bacteria. ^1^ T1 = control, T2 = 1% garlic essential oil (*w*/*w*), T3 = 1% rosemary essential oil (*w*/*w*), T4 = 0.5% chipotle pepper oleoresin (*w*/*w*), T5 = 1% garlic essential oil + 0.5% chipotle pepper oleoresin (*w*/*w*) and T6 = 1% rosemary essential oil + 0.5% chipotle pepper oleoresin (*w*/*w*). ND = not detected, below the detection limit. ^a,b,c^ = different literals in the same row indicate significant difference (*p* < 0.05) between treatments. ^A,B,C^ = different literals in the same column indicate significant difference (*p* < 0.05) over time.

## Data Availability

Data are contained within the article.
